# Cell membrane-anchored MUC4 promotes tumorigenicity in epithelial carcinomas

**DOI:** 10.18632/oncotarget.13122

**Published:** 2016-11-04

**Authors:** Pengpeng Xia, Agnes Hakyung Choi, Zengping Deng, Yuqian Yang, Jing Zhao, Yiting Wang, Philip R. Hardwidge, Guoqiang Zhu

**Affiliations:** ^1^ College of Veterinary Medicine, Yangzhou University, Yangzhou, China; ^2^ Jiangsu Co-Innovation Center for Prevention and Control of Important Animal Infectious Diseases and Zoonoses, Yangzhou, China; ^3^ Nanjing Hospital of T.C.M, Nanjing, China; ^4^ College of Veterinary Medicine, Kansas State University, Manhattan, KS, USA

**Keywords:** MUC4, carcinoma, epithelial, tumorigenicity

## Abstract

The cell surface membrane-bound mucin protein MUC4 promotes tumorigenicity, aggressive behavior, and poor outcomes in various types of epithelial carcinomas, including pancreatic, breast, colon, ovarian, and prostate cancer. This review summarizes the theories and findings regarding MUC4 function, and its role in epithelial carcinogenesis. Based on these insights, we developed an outline of the processes and mechanisms by which MUC4 critically supports the propagation and survival of cancer cells in various epithelial organs. MUC4 may therefore be a useful prognostic and diagnostic tool that improves our ability to eradicate various forms of cancer.

## INTRODUCTION

Mucins are high-molecular-weight epithelial glycoproteins that can be subcategorized into secretory, gel-forming, or membrane-anchored [1]. Mucins serve as the primary line of defense against extracellular disruptions, including pathogen infection, pH changes, and ion influx [1–5].

MUC4 is a transmembrane mucin that belongs to the membrane-anchored class of mucins, and its expression is tissue-dependent [6]. MUC4 is also a tumor-associated antigen, whose overexpression is observed in various epithelial malignancies, such as pancreatic adenocarcinoma and breast, lung, ovarian, and prostate cancer. The methylation and histone deacetylation profiles of the MUC4 5′-untranslated region (UTR) suppress MUC4 expression [7, 8]. The presence of MUC4 in absorptive, ciliated, and goblet cells suggests it can be both membrane-anchored and secreted [6]. When mucous layer reinforcement is needed, MUC4 undergoes proteolytic cleavage at a conserved putative GDPH proteolytic cleavage site to become a secreted, non-gel-forming mucin [9].

The multiple cellular functions directed by the sequence, structure, and glycosylation of MUC4 suggest it promotes the progression of various forms of epithelial carcinoma [3, 10]. MUC4 expression has an inverse relationship with the survival rates of epithelial carcinoma patients [11, 12]. Thus, MUC4 represents a potential diagnostic and prognostic target.

## MUC4 STRUCTURE AND FUNCTIONS

**Figure 1:**
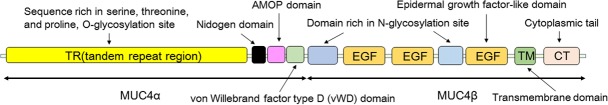
A schematic view of the MUC4 protein architecture MUC4 protein is composed of two subunits, MUC4α and MUC4β. MUC4α is the extracellular and hyper-glycosylated subunit (500-850 kDa), and MUC4β is the membrane-bound subunit that contains EGF-like domains and many N-glycosylation sites (80-90 kDa).

MUC4, which is homologous to rat sialomucin complex (SMC)/Muc4, is abundantly in carbohydrates [13]. The hundreds of carbohydrates bound to MUC4 are O-glycosidically linked to the protein backbone, which possesses tandem repeat peptides primarily composed of serine, threonine, and proline [6]. Carbohydrate moieties, primarily *N*-acetylglucosamine, galactose, fucose, *N*-acetylgalactosamine, and sialic acid, are the most prominent branches of MUC4. The *MUC4* gene is located on chromosomal locus *3q29* [14], and encodes the large 930 kDa polypeptide precursor apomucin, which is composed of subunits MUC4α and MUC4β [15]. MUC4α is the 500-850 kDa, extracellular, hyper-glycosylated subunit; MUC4β is the 80-90 kDa, membrane-bound subunit that contains EGF-like domains and many *N*-glycosylation sites (Figure 1). The MUC4 precursor is enzymatically separated to form the final heterodimeric, bi-functional, cell surface glycoprotein, which varies in size from 4,468 to 8,468 amino acid residues [15].

The transmembrane domain of MUC4 allows the glycoprotein to participate in intracellular processes and interact with extracellular molecules and receptors via its large, mucin-like N-terminus protruding from the cell surface [16, 17]. In contrast to other membrane-anchored mucins, MUC4 contains a von Willebrand factor type D domain, a nidogen domain, and an adhesion-associated domain (Figure 1) [18, 19]. MUC4 has twenty-four variant forms, resulting from alternative splicing events that lead to differences in glycosylation and the variable number of tandem repeats (VNTR) domain [6, 20–23]. *O*-glycosidically linked oligosaccharides are distinguished by their core type, backbone type, and peripheral structures, all of which contribute to the filamentous conformation of the MUC4 extracellular domain [6]. In addition, *O*-linked carbohydrates contain sialic acid and sulfate residues that add a negative charge to the N-terminus of the glycoprotein. In the filamentous conformation, the extracellular domain of MUC4 extends 1.12-2.12 μm above the cell surface and interacts with extracellular molecules that bypass the initial mucosal barrier covering the epithelium [24].

The MUC4 ectodomain, which possesses the filamentous structure of *O*-glycosylated tandem repeats, contributes to the protective mucous layer by forming a physical barrier involved in growth and survival signaling. These MUC4 functions are exploited by various carcinomas to promote the propagation and survival of tumor cells [25]. The high concentration of carbohydrates in MUC4, whose characteristic filamentous structure extends well above the glycocalyx, is crucial for glycoprotein survival. MUC4-bound oligosaccharides facilitate resistance to degradation in the gastrointestinal tract, in addition to their role in mediating the recognition of extracellular lectins and antibodies [6]. The MUC4 extracellular domain, which is represented by the heavily *O*-glycosylated and filamentous apomucin, is responsible for navigating the extracellular environment via ligand binding [26, 27].

ErbB2, a cognate receptor tyrosine kinase also referred to as HER2 in humans and neu in rodents, binds to the EGF-like motifs of MUC4 to facilitate signal transduction, cell proliferation, and cell survival [4, 9, 24, 26, 28]. This *cis* interaction is restricted to cellular surfaces on which signal transduction occurs via the formation of an ErbB2-MUC4 complex [24, 27, 29]. MUC4 binding to ErbB2 starts with ErbB3 binding to neuregulin (NRG), followed by the formation of a heteromeric complex between NRG and ErbB2. The formation of this complex leads to the phosphorylation-mediated activation of ErbB2, which then binds to MUC4, forming a tetrameric MUC4-ErbB2-ErbB3-NRG complex. MUC4 stabilizes this complex by preventing its internalization [16, 24, 30, 31]. MUC4 can initiate and/or potentiate downstream MAPK signaling associated with differentiation and proliferation by imposing a protection mechanism for cell polarization [32].

**Figure 2:**
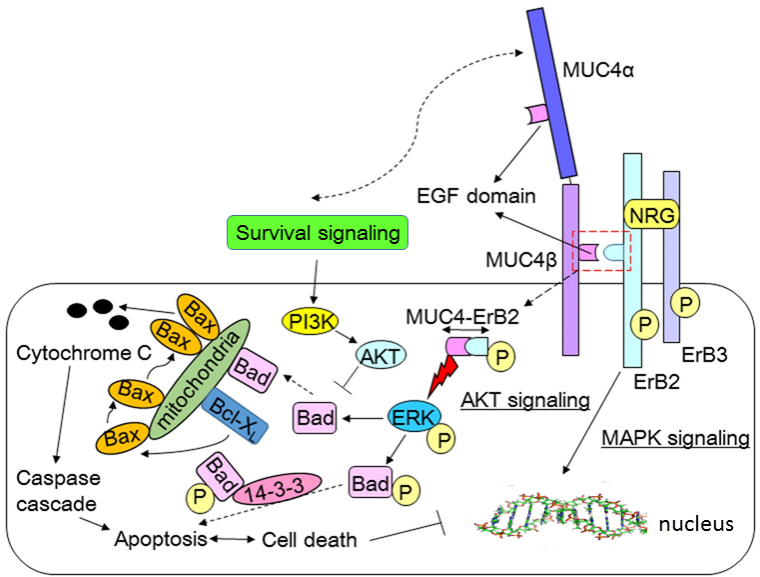
The functions and roles of MUC4 in various signaling transduction pathways ErbB3 binds with neuregulin (NRG), followed by the formation of a heteromeric complex between NRG and ErbB2. The formation of this complex leads to the phosphorylation-mediated activation of ErbB2. The activated ErbB2 binds to the EGF-like motifs of MUC4, forming a tetrameric MUC4-ErbB2-ErbB3-NRG complex. The MUC4-ErbB2-ErbB3-NRG complex activates the protein kinase B (PKB)/Akt and mitogen-activated protein kinase (MAPK)/extracellular signal-regulated kinase (ERK) pathways to induce cell proliferation and inhibit apoptosis. The formation of the tetrameric MUC4-ErbB2-ErbB3-NRG complex leads to the hyper-phosphorylation of ErbB2. That phosphorylation enables the downstream activation of the phosphoinositide-3 kinase (PI3K)-Akt and Ras-ERK pathways, which induce a loss of cell polarity in tumor cells. In addition to activating growth and survival signals in pancreatic cancer cells, MUC4 deactivates pro-apoptotic proteins. In prostate cancer cells, ErbB2/HER2 activates ERK to phosphorylate and deactivate the protein Bad, which is then unable to deactivate the anti-apoptotic proteins Bcl-2 and Bcl-XL. In response to gemcitabine, MUC4 promotes the phosphorylation of Bad via its stimulation of HER2 and activation of ERK. These effects ultimately lead to the suppression of both cytochrome C release and cancer cell apoptosis

Along with cell protection, MUC4 is also involved in extracellular factor-cell communication, cell proliferation, and adhesion. Its roles are most notably intertwined with the molecular mechanisms underlying the neoplastic progression and metastasis of various forms of epithelial carcinoma (Figure 2). MUC4 functions are initiated upon the activation of p27(kip), a cell cycle inhibitor [26, 33]. The MUC4-ErbB2-ErbB3-NRG complex activates the protein kinase B (PKB)/Akt and mitogen-activated protein kinase (MAPK)/extracellular signal-regulated kinase (ERK) pathways to induce cell proliferation and inhibit apoptosis (Figure 2) [33]. The formation of the tetrameric MUC4-ErbB2-ErbB3-NRG complex leads to the hyper-phosphorylation of ErbB2. This phosphorylation enables the downstream activation of the phosphoinositide-3 kinase (PI3K)-Akt and Ras-ERK pathways, which induce a loss of cell polarity in tumor cells. In addition, the increased activation of these pathways results in the transcriptional activation of cyclin D1, leading to increased cell proliferation [24, 26]. MUC4 also facilitates cellular adhesion and subsequent binding to the endothelium and activates immunosuppressive responses to tumor cells. Decreased MUC4 expression is associated with reduced cell proliferation and motility and increased cellular aggregation [34].

MUC4 levels inversely correlate with the 5′-UTR DNA methylation level in various cancer cell lines. *MUC4* gene expression depends on the methylation status of CpG motifs near the transcriptional start site of *MUC4* [7, 8]. In addition to DNA methylation, histone modification suppresses *MUC4* expression. Histones correlated with MUC4 expression are commonly deacetylated in pancreatic and gastric epithelial cancer cell lines [7, 8, 31, 35].

## FACTORS REGULATING MUC4 EXPRESSION

Overexpression of MUC4 in human tumor cells promotes anti-adhesive functions and represses the anti-tumor functions of the immune system [36]. MUC4 expression is up-regulated by various factors. This includes PEA3, an Ets family member (E26 transcription factor) that is involved in proliferation, differentiation, and transformation. The IFN-γ inflammatory pathway increases MUC4 expression via STAT-1 up-regulation [37, 38]. Transforming growth factor (TGF)-β participates in pancreatic carcinogenesis by activating MUC4 expression via the MAPK, PI3K, and protein kinase A (PKA) signaling pathways. All-trans-retinoic acid (RA) treatment increases MUC4 expression via RA receptor-α and TGF-β2 [26, 27, 32, 38–42].

Additional potent promoters of MUC4 include the transcription factors hepatocyte nuclear factor (HNF)4α11, forkhead box A (FOXA)1/FOXA2, GATA-4/-5/-6, and caudal-related homeobox (CDX)-1/-2 [43]. These factors stimulate cell differentiation in gut endoderm- and pancreas-derived tissues during embryonic development [15]. This differentiation network is involved in pancreatic development and is thought to increase mucin expression during carcinogenesis in a similar manner to its activities during embryonic growth.

MUC4 levels follow the cyclic expression patterns of hormones associated with the menstrual cycle [34]. MUC4 is overexpressed in cervical cancer tissues, with the highest level of MUC4 expression in high-grade dysplasia. The differences in MUC4 expression patterns between dysplastic and normal endocervical epithelia may be useful as a diagnostic marker for predicting cervical cancer [2, 44–46]. MUC4 expression is a marker of good prognosis in upper aerodigestive tract carcinomas, but a marker of poor prognosis in ovarian carcinomas [47, 48].

Metastasis is induced by MUC4, which triggers the dissociation of tumor cells from the primary tumor site by blocking surface adhesion molecule binding, integrin-mediated cell adhesion, and homotypic cell-cell interactions [49]. Elevated MUC4 expression increases aggression of breast cancer, including decreased binding of breast cancer tissues to extracellular structures such as laminin, fibronectin, and collagen and reduced cell-cell interactions among these structures [50, 51]. Furthermore, the expression of MUC4 in breast cancer cells decreases apoptosis 5-10-fold relative to nonmalignant breast cells. This demonstrates that the primary function of MUC4 is to promote tumorigenesis by suppressing apoptosis via alterations of signal transduction processes ranging from modification of cell surface interaction sites to regulation of protein synthesis [25, 47]. In breast cancer, the post-transcriptional regulation of MUC4 is lost when cancer cells become unresponsive to TGF signals, most likely as a result of ErbB2 overexpression [27, 31, 47, 51].

## CHANGES IN MUC4 EXPRESSION IN CANCER CELLS

Epithelial invasion is triggered by the anti-adhesive activities of MUC4, which leads to the disassociation of tumor cells from the primary tumor site [49]. The anti-recognition activity of MUC4 promotes tumor growth by enabling the evasion of both immune surveillance and apoptosis suppression. As a result, tumor propagation is accelerated, and tumor cells migrate to the bloodstream. These events are facilitated by the sialyl-oligosaccharides on MUC4 and ultimately result in tumor cell invasion and endothelial transmigration. Cancer stem cells and MUC4 contribute to cancer relapse and progression by assisting in the initiation, growth, and recurrence of various carcinomas [52].

As a consequence of the stress induced by carcinogenesis and malignant cellular transformation, a loss of cell polarity occurs in association with activation of cellular proliferation and survival systems [2]. The process underlying the onset and maintenance of cell polarity loss is termed the epithelial-mesenchymal transition (EMT) [53, 54]. MUC4 stimulates the EMT via its effect on β-catenin, which is known to promote cell-cell adhesions through its association with cadherin complexes in the cell membrane. β-catenin also acts together with TCF as a transcriptional co-activator to induce the EMT in tumor cells upon its translocation to the nucleus. The EMT is initiated by a decrease in E-cadherin expression and an increase in vimentin filaments. These changes are followed by cellular transformation from an immotile, polarized state to a motile, mesenchymal state. Cells also adopt a spindle-like conformation rather than their original cobblestone-like morphology, and lose the expression of various differentiation markers and cell-cell junction proteins. All of these factors contribute to an overall increase in migratory capacity, a decrease in the expression of epithelial markers such as E-cadherin, and an enhancement of local invasiveness [55].

In tumor cells accustomed to long-term EMT, MUC4 freely binds with molecules that normally do not appear on the non-apical surface, which contributes to a sustained state of reduced cell polarity [56]. For example, ErbB2 is normally sequestered in epithelial cells by the MUC4-ErbB2 complex, but upon the loss of cell polarity and tight junctions, ErbB3 becomes accessible for binding to ErbB2. This leads to the activation of the ErbB2 signaling pathway and to the reduced binding of ErbB2-specific antibodies [57]. The EMT is promoted and maintained via the ErbB2-mediated disruption of the Par complex. This leads to the loss of function of cellular tight junctions, thus inducing unresponsiveness to cell-cell/cell-extracellular factor communication [2].

MUC4 expression in normal pancreatic tissue is typically undetectable, but as healthy cells transform into carcinogenic cells, MUC4 expression increases (Table 1) [58]. In the case of ovarian cancer, the level of MUC4 is actually not predictive of the disease [59]. MUC4 expression is associated with the malignant transformation of ovarian epithelial cells into cancer cells. Therefore, a reduction in MUC4 expression during the late stage of ovarian cancer indicates a greater likelihood of survival [52, 59]. This is in contrast to the majority of forms of epithelial carcinoma, which show abnormally high rates of MUC4 expression that increase with disease progression. The above trends suggest a possible relationship between MUC4 expression and the development of metastasis in ovarian epithelial cells. The specific and aberrant patterns of MUC4 expression during the progressive stages of various forms of epithelial carcinoma implicate MUC4 as a novel potential biomarker and therapeutic target [15].

**Table 1:** MUC4 expression in different cancers

	Cancer tissue	Normal tissue	Detection method
Pancreatic cancer	High***/High*	Under detectable level	Q, R, I, W, C etc [37, 89, 90]
Ovarian cancer	High**/High*	-	R, I, W etc [49, 52, 60]
Breast cancer	High**	Under detectable level	Q, R, I, W etc [51, 91, 92]
Lung cancer	High***/High*	Detectable/High*	R, I, W etc [24, 56, 93]
Colon cancer	High***	High**	Q, R, I, W etc [71, 94]
Prostate cancer	High**	High*	Q, R, I etc [95–97]
Head-neck cancer	High*	Detectable	I, W etc [77, 98–100]
Cervical cancer	High***	Detectable/High*	I, S etc [44–46]

*** The expression of MUC4 can be used as a prognostic marker; ** the expression of MUC4 is very high; * the expression of MUC4 is higher than the threshold for detection; / MUC4 expression changes at different stages of cancer progression. Q-quantitative real-time PCR; R-real time PCR; W-western blot; I-immunohistochemistry; S-Semi-quantitative RT-PCR; C-Chromatin immune-precipitation assays (ChIP) etc.

Tumor cells overexpress aberrantly glycosylated mucins that are secreted into the bloodstream and can serve as tumor markers [30]. Alterations in mucin glycosylation during cancer most likely results from glycosyltransferase activity or post-translational modifications in the specific apomucin residues that can be glycosylated. As a result of these alterations, mucins become more easily detectable in ovarian and breast cancer patients. Whereas ovarian cancer cells express several different mucin glycoproteins during metastasis (MUC2, MUC3 and MUC5B), non-malignant ovarian cells do not express these mucins. In completely metastatic ovarian cancer cells, aberrant expression of MUC4 implies mucins are correlated with the malignant transformation of cells during specific stages of metastatic progression [52, 60].

In pancreatic cancer, MUC4 expression is increased, especially throughout the stages of tumor development leading to carcinoma [58]. MUC4 or apomucin transcription is induced in pancreatic intraepithelial neoplasia lesions and intraductal papillary mucinous neoplasms. Increased expression of *MUC4* induced by mutant K-ras (a small GTPase of the Ras superfamily) correlates with the activation of the ERK, JNK, and NF-κB signaling pathways. This transcriptional up-regulation leads to increased expression of MUC4 in pancreatic adenocarcinoma [38]. *MUC4* is the most differentially expressed gene in pancreatic ductal adenocarcinoma (PDC), and its expression level differed between each stage of cancer progression [58, 61]. In addition, the MUC4-ErbB2-ERK pathway has been shown to contribute to the characteristic resistance of PDC to gemcitabine by inhibiting the activation of intrinsic apoptosis in cancer cells [62, 63].

MUC4 associates with the host receptor of enterotoxigenic *Escherichia coli* [64–68]. Moreover, *E. coli* strains from phylogroup B2 harboring a pks+ island can produce a peptide-polyketide hybrid compound termed colibactin, which can trigger premature and transmissible senescence in mammalian cells, resulting in colon cancer [69]. MUC4 promotes intestinal cell proliferation during tumorigenesis, and mice deficient in MUC4 exhibited reduced tumor burden compared with WT mice [70]. Therefore, it is necessary to investigate the relationship between MUC4 and colibactin, and focusing on MUC4 might reveal a new strategy to prevent colon cancer [60, 71].

## MUC4 AND CANCER TREATMENT

Thymoquinone (TQ) down-regulates MUC4 expression via the proteasomal pathway, and induces apoptosis in pancreatic cancer cells by activating the c-Jun N-terminal kinase (JNK) and p38 MAPK pathways [72]. Both of these pathways are stimulated by MUC4, and are used by cancer cells to proliferate, to defend against the immune response, and to promote the invasion of distal epithelial tissue [72]. A decrease in the MUC4 level increased the rate of apoptosis in cancer cells, decreased their motility, and reduced overall epithelial cell migration [72–76]. MUC4 overexpression is correlated with the proliferation and cellular senescence of head and neck squamous cell carcinoma (HNSCC) cells, and down-regulating MUC4 expression may serve as a promising therapeutic approach for treating HNSCC patients [77]. MUC4 down-regulation improves the efficacy of gemcitabine to eradicate pancreatic carcinoma cell masses [78].

In addition to decreasing cell migration and motility, down-regulation of MUC4 expression hampers the evasion of cancer cells from apoptosis and immune surveillance [72]. The MUC4 promoter contains many Smad-binding sites. TGF-β is a pleiotropic cytokine that is involved in gene expression through its activation of Smad proteins [27, 79]. In the early phases of carcinogenesis, TGF-β inhibits cellular growth, but in later phases, it contributes to the progression of tumor metastasis. TGF-β also suppresses MUC4 expression post-transcriptionally through the proteasomal pathway, and activates the JNK and p38 MAPK pathways[79].

These findings regarding MUC4 suppression/repression suggest the potential of this glycoprotein as a key target of successful cancer therapy. MUC4-based vaccines induced strong antigen-specific immune responses in mice [80]. A dendritic cell (DC)-based vaccine, using cells transduced with an adenovirus encoding the universal DR-restricted Th helper epitope (PADRE) combined with HLA-A1(/A2) restricted epitopes, may be a potential strategy for the immunotherapy of MUC4-associated tumors [81].

As mitochondrial number increases, apoptosis rates normally increase [10]. This is because mitochondria release factors that induce apoptosis, primarily cytochrome *c*. However, mini-MUC4-transfected pancreatic cancer cells showed increased mitochondrial mass without the expected enhancement of apoptosis. In poorly differentiated pancreatic cell lines, MUC4 overexpression likely leads to the sequestration of apoptogenic factors in the mitochondrial inter-membrane space due to changes in the polarization state of the mitochondrial membrane. This inhibits apoptosis without preventing the increase in the number of mitochondria [10, 31, 63, 82].

In addition to activating growth and survival signals in pancreatic cancer cells, MUC4 deactivates pro-apoptotic proteins by inducing the unresponsiveness of cancer cells to apoptotic signals [49, 83]. In prostate cancer cells, ErbB2/HER2 activates ERK to phosphorylate and deactivate Bad, which is then in turn unable to deactivate the anti-apoptotic proteins Bcl-2 and Bcl-X_L_. These events suppress the responses to intrinsic mitochondrial apoptotic pathway activity (Figure 2) [83]. In response to gemcitabine, MUC4 promotes the phosphorylation of Bad via its stimulation of HER2 and its activation of ERK. These effects ultimately lead to the suppression of both cytochrome C release and cancer cell apoptosis [63, 82–84].

MUC4 overexpression increases the phosphorylation and activation of a non-receptor tyrosine kinase termed focal adhesion kinase (FAK), as well as the Akt and ERK pathways. These events promote downstream HER2 signaling and alter the actin network to increase the motility of ovarian cancer cells. Other factors contributing to the motility of MUC4-transfected cells are filopodia, lamellipodia, and microspikes, which assist in propelling cell movement [74]. In pancreatic cancer cells, MUC4 up-regulation causes ultra-structural changes that promote tumorigenicity via proliferation and modified interactions with the extracellular matrix [30].

In ovarian cancer development, the loss of cell polarity enables atypical protein-protein interactions, allowing MUC4 to activate ErbB2/HER2 expression. This was determined by silencing MUC4 using antisense RNA, which results in a decrease in ErbB2/HER2 expression, which leads to decreases in the ovarian cancer cell motility rate and overall invasiveness [26, 31, 52, 74, 85, 86].

## CONCLUSIONS

The multiple functions of MUC4 originating from its abundant polymorphic capabilities, its glycosylation, the flexibility of its protein backbone structure, and the repeats in its VNTR region are among the primary influencers of cancer cell growth [4, 31]. As components of cellular signal transduction mechanisms, cell propagation, apoptosis pathways, and anti-adhesive and EMT regulation systems, the functions of MUC4 are understandably highly controlled during tumorigenesis and are hijacked by tumor cells to promote proliferation, dissociation, and metastasis to distal regions [1, 17, 25, 34, 48].

The expression of MUC4 is tissue and organ specific, and alterations to its mRNA expression, protein backbone structure, or glycosylation profile lead to the malignant transformation of organ tissue cells. The distribution of MUC4 changes in the stratified epithelium, and its expression is aberrant in cancers of the upper aerodigestive tract. However, in such forms of cancer, the presence of MUC4 indicates a more favorable prognosis. Additionally, the distribution of overexpressed MUC4 is altered in muco-epidermoid cancers of the salivary gland. In both low- and high-grade forms of these cancers, MUC4 expression is correlated with an increased survival rate and a reduced recurrence rate. However, in lung adenocarcinoma, particularly in stage 1A cases, high expression of MUC4 indicates a reduced survival rate compared to similar cases with low MUC4 expression [25].

In biliary tract cancer, there is a roughly 1.9-fold increase in MUC4 mRNA levels, and increased MUC4 expression is associated with a decreased survival rate among these patients [2, 87]. Although MUC4 is undetectable in normal bile duct cells, its expression is detectable in the larger bile ducts of intra- and extrahepatic cholangiocarcinomas, as well as ductal adenocarcinomas of the pancreas [88].

Understanding the MUC4 interactome may improve the evaluation of specific predictive markers and the selection of the MUC4 pathway inhibitors in controlling cancer progression.
